# U–Th whole rock data and high spatial resolution U–Th disequilibrium and U–Pb zircon ages of Mt. Erciyes and Mt. Hasan Quaternary stratovolcanic complexes (Central Anatolia)

**DOI:** 10.1016/j.dib.2020.105113

**Published:** 2020-01-11

**Authors:** Bjarne Friedrichs, Axel K. Schmitt, Lucy McGee, Simon Turner

**Affiliations:** a Institute of Earth Sciences, Heidelberg University, Germany; b Department of Earth and Planetary Sciences, Macquarie University, Sydney, Australia; c Department of Earth Sciences, University of Adelaide, Australia

**Keywords:** U-series dating, Zircon, Secondary Ion Mass Spectrometry (SIMS), Central Anatolian Volcanic Province (CAVP), Cappadocia, Turkey, SIMS, Secondary Ion Mass Spectrometry, MC-ICP-MS, Multi-Collector Inductively Coupled Plasma Mass Spectrometry

## Abstract

Thirty-eight lava and pyroclastic samples were collected from Mt. Erciyes and Mt. Hasan, the two largest stratovolcanic complexes of the Central Anatolian Volcanic Province in Turkey. More than 1000 zircon crystals were dated by Secondary Ion Mass Spectrometry (SIMS) applying U–Th disequilibrium and U–Pb methods. Model ages were calculated from zircon ^230^Th–^238^U–^232^Th isotopic compositions in combination with U–Th whole rock data of digested lava samples generated by Multi-Collector Inductively Coupled Plasma Mass Spectrometry (MC-ICP-MS). Middle and Late Pleistocene ages dominate the dataset, but are complemented by both older (predominantly Early Pleistocene) and younger (Holocene) ages. U–Th disequilibrium and U–Pb zircon data provide maximum eruption ages that can be further specified by (U–Th)/He geochronology (zircon double dating). Additionally, these data are important to constrain the longevity and size of magmatic systems, and their potential for reactivation leading to potentially hazardous eruptions.

**Table:** Specifications Table

Subject	Geochemistry and Petrology
Specific subject area	Geochronology, Geochemistry
Type of data	Tables
How data were acquired	Multi-Collector Inductively Coupled Plasma Mass Spectrometry (MC-ICP-MS); Nu Instruments Nu Plasma; Macquarie University, Sydney, Australia Secondary Ion Mass Spectrometry (SIMS); CAMECA ims 1280-HR; Heidelberg University, Germany
Data format	MC-ICP-MS: U–Th whole rock isotope data in *.xlsx format (Supplementary Table 1) SIMS: U–Th–Pb zircon data in *.xlsx format (corrected for relative sensitivity and Th disequilibrium; Supplementary Tables 3 and 4)
Parameters for data collection	MC-ICP-MS: Lava bulk rock samples were powdered, spiked, and digested. U and Th were extracted by column separation. SIMS: Zircon crystals were separated from lava and composite pumice samples, rinsed in HF, and pressed in Indium (rim analyses). Selected crystals were re-mounted in epoxy resin and polished (interior analyses).
Description of data collection	MC-ICP-MS: U and Th concentrations and isotope ratios were determined by separate isotope dilution analyses. SIMS: ^238^U^16^O^+^, ^232^Th^16^O^+^, and ^230^Th^16^O^+^ were analysed simultaneously in multi-collection mode. ^204^Pb^+^, ^206^Pb^+^, ^207^Pb^+^, ^208^Pb^+^, ^232^Th^+^, ^238^U^+^, ^238^U^16^O^+^, and ^238^U^16^O_2_^+^ were analyzed sequentially in single-collection mode.
Data source location	Mt. Erciyes and Mt. Hasan stratovolcanic complexes (Central Anatolia) as plotted in Fig. 1 and reported in Table 1.
Data accessibility	With the article

**Table:** 

**Value of the Data** • U–Th disequilibrium and U–Pb zircon crystallization ages define maximum eruption ages for a comprehensive sample set of Mt. Erciyes and Mt. Hasan volcanic systems (Central Anatolia). • The dataset provides a basis for the study of magma chamber processes and related volcanic hazard assessments to petrologists and volcanologists, respectively. • Zircon crystallization ages can be employed for ^238^U–^230^Th disequilibrium corrections to enhance (U–Th)/He geochronology. • Age spectra and the combination of rim and interior analyses can help to constrain the longevity and size of magmatic systems.

## 1 Data

**Fig. 1:**
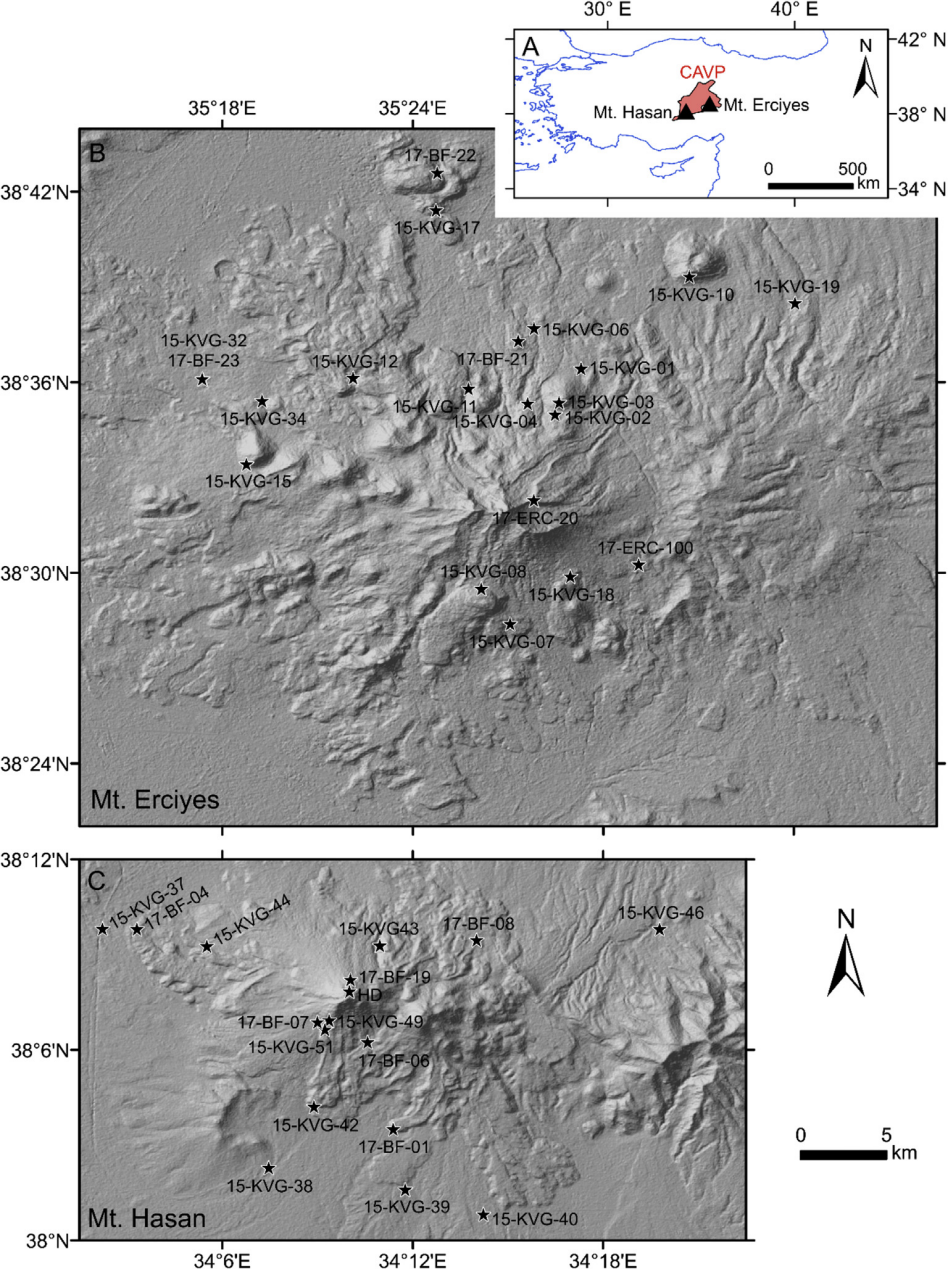
Overview map with the Central Anatolian Volcanic Province (CAVP) in Turkey (A) and sample locations at Mt. Erciyes (B) and Mt. Hasan (C) on a digital elevation model [1] at similar scales.

An overview map and sample locations plotted on a digital elevation model [1] are given in Fig. 1. Descriptions and coordinates for 38 andesitic to rhyolitic lava and pyroclastic samples of Mt. Erciyes and Mt. Hasan Quaternary stratovolcanic complexes are provided in Table 1. U–Th whole rock isotope data for six lava samples are reported in Supplementary Table 1. Equipoints employed for U–Th disequilibrium age calculations are stated in Supplementary Table 2. High spatial resolution U–Th and U–Pb zircon geochronological data for 1136 crystals are presented in Supplementary Table 3 (U–Th) and Supplementary Table 4 (U–Pb).

**Fig. 2:**
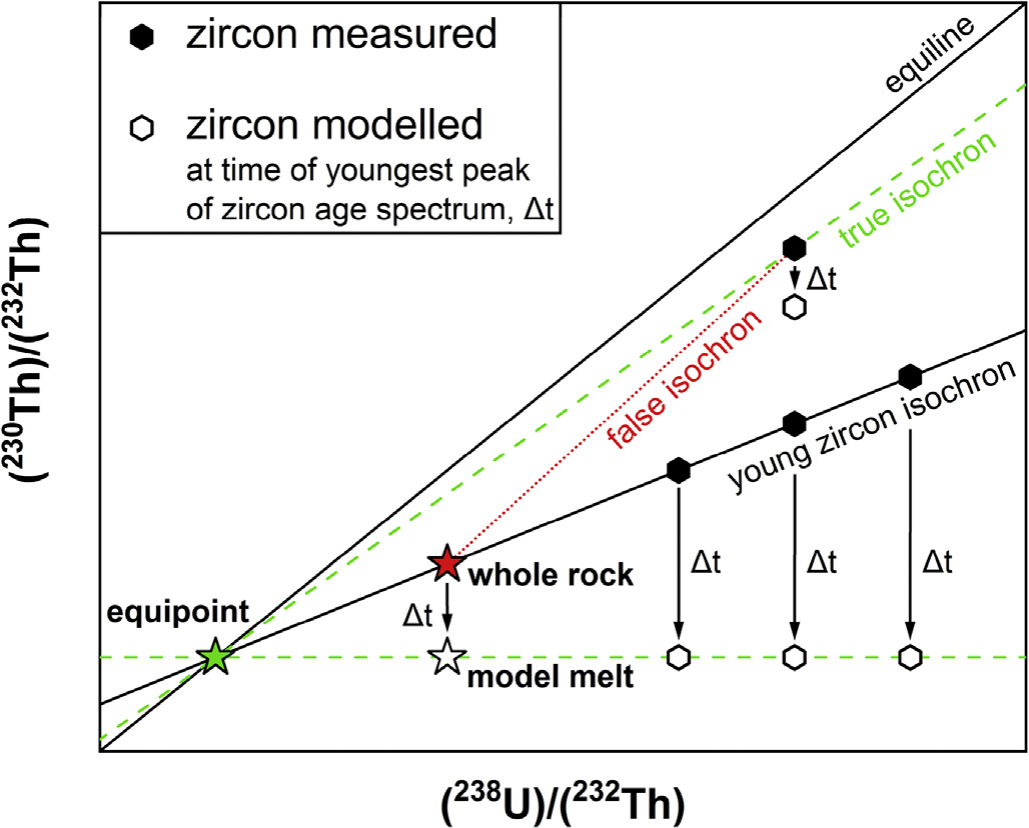
Schematic illustration of calculation of an equipoint (green star; Supplementary Table 2) based on a measured whole rock (^238^U)/(^232^Th) (red star; Supplementary Table 1) and the corresponding model melt (^230^Th)/(^232^Th) at the time of the youngest peak of the zircon age spectrum (Δt; white star); this peak was identified as the youngest maximum in the probability density function of individual zircon isochron slopes. The projection of the model melt to an equipoint on the equiline simulates identical melt compositions for each zircon at the time of its crystallization and precludes false isochrons (red dotted line). U–Th disequilibrium ages presented in Supplementary Table 3 are thus based on such equipoints.

**Table 1:** Sample descriptions and locations in WGS84 coordinate system.

Volcano	Sample	Sample Type	Type of Deposit	Location	Longitude [°E]	Latitude [°N]	Altitude [m]
Mt. Erciyes	15-KVG-01	Composite pumice	Pyroclastic flow	SE’ Hacılar	35.48838	38.60710	1777
Mt. Erciyes	15-KVG-02	Composite pumice	Fall-out (Perikartın)	NE’ Perikartın Dome	35.47463	38.58301	2346
Mt. Erciyes	15-KVG-03	Dacite lava	Lava dome	Lifos Hill summit	35.47684	38.58928	2438
Mt. Erciyes	15-KVG-04	Rhyolite lava	Lava dome	Perikartın Dome	35.46033	38.58882	2165
Mt. Erciyes	15-KVG-06	Composite pumice	Fall-out (Karagüllü)	NE’ Karagüllü Dome	35.46366	38.62828	1518
Mt. Erciyes	15-KVG-07	Composite pumice	Fall-out (Dikkartın)	Dikkartın quarry	35.45111	38.47318	2186
Mt. Erciyes	15-KVG-08	Rhyolite lava	Lava dome	Dikkartın Dome	35.43597	38.49153	2561
Mt. Erciyes	15-KVG-10	Dacite lava	Lava dome	Ali Dağ Dome	35.54519	38.65537	1601
Mt. Erciyes	15-KVG-11	Rhyolite lava	Lava dome	Karagüllü Dome	35.42927	38.59659	1928
Mt. Erciyes	15-KVG-12	Dacite lava	Lava flow	Şeyharslantepe	35.36866	38.60228	1765
Mt. Erciyes	15-KVG-15	Dacite lava	Lava dome	Gökdağ Dome	35.31264	38.55700	1844
Mt. Erciyes	15-KVG-17	Dacite lava	Lava dome	S′ Yılanlı Dağ Dome	35.41218	38.69023	1306
Mt. Erciyes	15-KVG-18	Dacite lava	Lava dome	Üç Tepeler	35.48270	38.49808	2579
Mt. Erciyes	15-KVG-19	Composite pumice	Ground surge (Valibabatepe ignimbrite)	E′ Zincidere	35.60070	38.64144	1426
Mt. Erciyes	15-KVG-32	Pumiceous xenolith	Scoria cone	W′ Kızılören (Karnıyarık)	35.28968	38.60165	1320
Mt. Erciyes	15-KVG-34	Pumiceous xenolith	Scoria cone	S′ Kızılören	35.32088	38.59018	1545
Mt. Erciyes	17-BF-21	Dacite lava	Lava flow	N′ Çarık Tepe	35.45537	38.62165	1738
Mt. Erciyes	17-BF-22	Dacite lava	Lava dome	NE’ Yılanlı Dağ Dome	35.41285	38.70983	1335
Mt. Erciyes	17-BF-23	Pumiceous xenolith	Scoria cone	W′ Kızılören (Karnıyarık)	35.28948	38.60163	1284
Mt. Erciyes	17-ERC-20	Dacite lava	Lava flow	E′ Mt. Erciyes summit	35.46356	38.53804	3378
Mt. Erciyes	17-ERC-100	Composite pumice	Fall-out (below paleosol)	Kayseri-Develi Road	35.51860	38.50429	2175
Mt. Hasan	15-KVG-37	Composite pumice	Pyroclastic flow (containing obsidian)	S′ Taşpınar	34.03711	38.16354	1060
Mt. Hasan	15-KVG-38	Bread crust bomb	Block-and-ash-flow	S′ Keçikalesi	34.12447	38.03805	1296
Mt. Hasan	15-KVG-39	Composite pumice	Pyroclastic flow (pumice-rich)	SE’ Karakapı	34.19608	38.02654	1333
Mt. Hasan	15-KVG-40	Bread crust bomb	Block-and-ash-flow	W′ Akçaören	34.23717	38.01357	1312
Mt. Hasan	15-KVG-42	Andesite lava	Lava flow	NE’ Keçikalesi	34.14816	38.07016	1532
Mt. Hasan	15-KVG-43	Obsidian lava	Lava flow	S′ Helvadere	34.18281	38.15471	2004
Mt. Hasan	15-KVG-44	Andesite lava	Lava flow	W′ Dikmen	34.09197	38.15438	1326
Mt. Hasan	15-KVG-46	Composite pumice	Pyroclastic flow	SW’ Kitreli	34.32973	38.16339	1541
Mt. Hasan	15-KVG-49	Andesite lava	Lava flow	Keçikalesi Plateau	34.15616	38.11538	2521
Mt. Hasan	15-KVG-51	Andesite lava	Lava flow	Keçikalesi Plateau	34.15395	38.11063	2374
Mt. Hasan	17-BF-01	Andesite lava	Lava flow	S′ Uluören	34.18982	38.05828	1469
Mt. Hasan	17-BF-04	Andesite lava	Lava flow	W′ Dikmen	34.05520	38.16335	1131
Mt. Hasan	17-BF-06	Andesite lava	Lava flow	N′ Karakapı	34.17633	38.10400	2118
Mt. Hasan	17-BF-07	Dacite block	Block-and-ash-flow	Keçikalesi Plateau	34.15002	38.11452	2276
Mt. Hasan	17-BF-08	Andesite lava	Lava flow	SW’ Yenipınar	34.23360	38.15758	1825
Mt. Hasan	17-BF-19	Andesite lava	Lava flow	N′ Mt. Hasan summit	34.16737	38.13680	2730
Mt. Hasan	HD [2]	Composite pumice	Fall-out	N′ Mt. Hasan summit	34.16679	38.13065	3160

## 2 Experimental design, materials, and methods

Uranium and Th isotopic ratios on bulk rock powders were determined at the U-series Research Laboratory at Macquarie University, Sydney, Australia. Approximately 0.2 g of powdered rocks were spiked with a ^236^U–^229^Th tracer solution and digested in a mixture of concentrated acids (HF–HNO_3_) in Teflon beakers at 190 °C for 66 hours. After digestion and dilution of the resultant solutions, U and Th were extracted from the rock matrixes using 4 ml columns of Biorad AG1-x8 anionic resin, introducing and eluting the samples in 7 N HNO_3_, and extracting the Th and U fractions in 6 N HCl and 0.2 N HNO_3_, respectively. Uranium and Th concentrations, determined by isotope dilution, and U–Th isotope ratios were measured separately on a Nu Instruments Nu Plasma MC-ICP-MS at Macquarie University. For U analyses, the New Brunswick Laboratory (NBL) synthetic standards U010 and U005a were used at regular intervals to assess the robustness of instrumental corrections and to monitor drift. For Th analyses, a standard-sample bracketing procedure for each sample analysed used the Th ‘U’ standard solution, and a linear tail correction for the ^232^Th tail on ^230^Th was applied. Sample 15-KVG-17 was duplicated as separate digestions that show good reproducibility in U and Th concentrations and activity ratios (see Supplementary Table 1 for data). One digestion of Table Mountain Latite (TML) was prepared and analysed with the samples, yielding data within error of reference values [3].

U–Th–Pb zircon analyses were performed at the HIP Laboratory at Heidelberg University. Samples were crushed and sieved (<125 μm) and zircon crystals were extracted by hydrodynamic separation and hand-picking. Adhering glass was dissolved by rinsing in cold 40% HF for ca. 3 minutes. Whole crystals were imbedded in indium (In) metal and their surfaces dated by U–Th disequilibrium methods (rim analyses) with a CAMECA ims 1280-HR SIMS at Heidelberg University. Crystals in equilibrium, within 1σ of (^230^Th)/(^238^U) = 1, were re-dated by U–Pb methods. Selected crystals were extracted from the In mounts, re-mounted in Epoxy resin, polished, and re-dated by U–Th disequilibrium and, if applicable, U–Pb methods (interior analyses). Analytical details are presented in Table 2, and data in Supplementary Table 3 (U–Th) and Supplementary Table 4 (U–Pb).

**Table 2:** Zircon U–Th–Pb analytical details.

Main categories	Specifications
Mounting types	Indium & Epoxy
Sample preparation and treatment before SIMS analysis	Work procedure (for Indium Mounts) 1. Standard imbedded, ground down & polished with SiC paper (FEPA# 800, 1200, 2400, 4000) & diamond paste (1 μm, 1/4 μm) 2. Samples imbedded, no grinding/polishing 3. Cleaned with EDTA + NH_3_, distilled water & methanol 4. Gold-coated (Quorum Q150T ES); Thickness of gold coating: 50 nm Work procedure (for Epoxy Mounts) 1. Ground down & polished to ∼20 μm depth with SiC paper (FEPA# 800, 1200, 2400, 4000) & diamond paste (1 μm, 1/4 μm) 2. Cleaned with EDTA + NH_3_, distilled water & methanol 3. Gold-coated (Quorum Q150T ES); Thickness of gold coating: 2 nm 4. Cathodoluminescence imaged at scanning electron microscope 5. Cleaned with EDTA + NH_3_, distilled water & methanol 6. Gold-coated (Quorum Q150T ES); Thickness of gold coating: 50 nm
Age calibration approach	Session-wise ThO^+^/UO^+^ relative sensitivity calibration using AS3 [4] & 91500 [5] reference zircons after [6]. For inter-session comparability, data presented in Supplementary Table 3 were re-calculated for secondary reference zircon AS3 to match unity. Session-wise UO_2_^+^/U^+^ vs. ^206^Pb^+^/U^+^ relative sensitivity calibration using AS3 [4] reference zircons.
Analytical conditions	U–Th conditions are described in [7]; U–Pb conditions in [8] Beam diameter: U–Th ∼40 μm (Köhler Ap.: 400 μm); U–Pb ∼20 μm (Köhler Ap.: 200 μm) Primary beam intensity: U–Th ∼10–70 nA; U–Pb ∼10–40 nA Mass resolution (M/ΔM): ∼4000 Raster conditions (during pre-sputtering): U–Th 10 μm, 10 s; U–Pb 15 μm, 20 s Note: U–Pb analysis spots were placed in U–Th analysis craters where both analyses were performed
Software to calculate ages	ZIPS 3.1.1
Method to calculate ages	U–Th: two-point isochron using zircon and equipoint (Fig. 2, Supplementary Table 2) U–Pb: ^207^Pb-corrected ^206^Pb/^238^U ages, disequilibrium-corrected using melt with Th/U = 3.148 for Mt. Erciyes and Th/U = 3.473 for Mt. Hasan samples (Supplementary Table 2)
Primordial lead model	Surface contamination ^207^Pb/^206^Pb = 0.847 [9]
Standards	AS3 (U–Th calibration, equilibrium; U–Pb calibration, 1099.1 Ma [4]), 91500 (U–Th calibration, equilibrium; U concentration, 81.2 ppm [5])
Secondary standards	U–Th: AS3; session-wise weighted mean values were: Session 2017_06: (^230^Th)/(^238^U) = 0.989; 1σ = 0.004; MSWD = 1.08; n = 73. Session 2017_09: (^230^Th)/(^238^U) = 1.018; 1σ = 0.004; MSWD = 1.07; n = 57. Session 2018_01: (^230^Th)/(^238^U) = 1.003; 1σ = 0.003; MSWD = 0.55; n = 78. Session 2018_07: (^230^Th)/(^238^U) = 1.025; 1σ = 0.005; MSWD = 0.51; n = 44. Session 2018_10: (^230^Th)/(^238^U) = 1.014; 1σ = 0.003; MSWD = 0.86; n = 119. Session 2019_07: (^230^Th)/(^238^U) = 1.001; 1σ = 0.007; MSWD = 0.95; n = 19. Session 2019_10: (^230^Th)/(^238^U) = 1.005; 1σ = 0.005; MSWD = 0.93; n = 49. U–Pb: 91500; session-wise (weighted mean) values were: Session 2017_06: ^206^Pb/^238^U Age = 1112 Ma; 1σ = 13 Ma; n = 1 (sample 15-KVG-19). Session 2017_06: ^206^Pb/^238^U Age = 1056 Ma; 1σ = 18 Ma; n = 1 (samples 15-KVG-32, 15-KVG-34). Session 2017_09: ^206^Pb/^238^U Age = 1060 Ma; 1σ = 36 Ma; MSWD = 0.01; n = 3. Session 2017_10: ^206^Pb/^238^U Age = 1274 Ma; 1σ = 91 Ma; n = 1 (unreliable). Session 2017_12: ^206^Pb/^238^U Age = 1086 Ma; 1σ = 47 Ma; n = 1 (sample 15-KVG-32). Session 2017_12: ^206^Pb/^238^U Age = 1101 Ma; 1σ = 19 Ma; MSWD = 0.05; n = 3 (samples 15-KVG-37, 15-KVG-39). Session 2018_01: ^206^Pb/^238^U Age = 1066 Ma; 1σ = 61 Ma; n = 1. Session 2019_01: ^206^Pb/^238^U Age = 1051 Ma; 1σ = 10 Ma; n = 1.
Decay constants	9.1577 × 10^−6^ a^−1^ for ^230^Th [10], 4.9475 × 10^−11^ a^−1^ for ^232^Th [11], 9.8485 × 10^−10^ a^−1^ for ^235^U, and 1.55125 × 10^−10^ a^−1^ for ^238^U [12].

## Appendix A Supplementary Tables 1–4

The following XLSX file contains Supplementary data to this article:
